# Association between triglyceride-glucose index and all-cause mortality in patients with congestive heart failure and atrial fibrillation

**DOI:** 10.3389/fcvm.2025.1476815

**Published:** 2025-04-03

**Authors:** Fuqiang Kan, Zewen Yang, Donglai Bao, Mingliang Tang, Ningning Ji

**Affiliations:** Department of Cardiology, Yiwu Central Hospital, The Affiliated Yiwu Hospital of Wenzhou Medical University, Yiwu, Zhejiang, China

**Keywords:** triglyceride-glucose index, all-cause mortality, congestive heart failure, atrial fibrillation, MIMIC-IV database

## Abstract

**Background:**

The role of the triglyceride-glucose (TyG) index in critically ill patients with congestive heart failure (CHF) and atrial fibrillation (AF), requiring intensive care unit (ICU) admission, remains unclear. This study aimed to investigate the association between the TyG index and the clinical prognosis of critically ill patients with CHF and AF.

**Methods:**

This retrospective observational cohort study utilized data from the Medical Information Mart for Intensive Care-IV (MIMIC IV2.2) database. Participants were categorized into four groups based on TyG index level. The primary outcome was hospital all-cause mortality. Multivariable logistic proportional regression analysis and restricted cubic spline regression were employed to assess the TyG index's association with hospital mortality in patients with CHF and AF. Sensitivity analysis included determining the TyG index's feature importance through subgroup analysis in different subgroups.

**Results:**

A total of 787 patients were included in the study, with hospital and ICU mortalities of 14.2% and 8.3%, respectively. Multivariate logistic regression analysis demonstrated that the TyG index was independently associated with an increased risk of hospital mortality (odds ratio (OR), 1.59 [95% confidence interval (CI) 1.15–2.19], *P* = 0.005) and ICU mortality [OR 1.9; (95% CI 1.28–2.83), *P* = 0.001] after adjusting for confounders. The restricted cubic spline regression model indicated a linear increase in the risks of in-hospital and ICU mortality with a higher TyG index. Sensitivity analysis revealed consistent effect sizes and directions in different subgroups, ensuring result stability.

**Conclusions:**

The results of our study suggest a significant association between the TyG index and hospital and ICU all-cause mortality in critically ill patients with CHF and AF. This finding implies that the TyG index could potentially serve as a valuable tool for identifying patients with CHF and AF at an elevated risk of all-cause mortality.

## Background

Heart failure (HF) is defined as the heart's inability to maintain adequate cardiac output without resorting to maladaptive compensatory mechanisms (1). Annually, over one million patients with HF are admitted to hospitals in the United States and Europe (2). Based on the findings of multiple heart failure registries, the in-hospital mortality rate for patients with acute heart failure (AHF) is 2%–8%. Within 3 months after discharge, the mortality rate reaches 10%–15%, with a readmission rate of 20%–30%. One year after discharge, the mortality rate reaches 20%–30%, and the readmission rate soars to 30%–50% (3–5). Due to the presence of common risk factors and the intricate interaction between them, atrial fibrillation (AF) and HF are observed to coexist in a significant proportion of patients, reaching up to 50% in some cases.1 An insightful analysis of participants from the Framingham Heart Study, who developed either new-onset AF or HF, uncovered compelling findings: 32% of patients diagnosed with HF had a prior history of AF, with an additional 30% being diagnosed with AF thereafter. Similarly, amongst those with new-onset AF, 8% had a pre-existing diagnosis of HF, while a further 28% were diagnosed with HF at a later stage (6). However, patients may already have symptomatic structural or functional cardiac abnormalities preceding CHF development (7, 8). Identifying prognostic-related biomarkers is crucial for early detection of high-risk patients, enabling more aggressive treatment measures.

Although the precise pathogenesis of CHF and AF remains unclear, insulin resistance (IR) plays a significant role (9). The triglyceride (TG)-glucose (TyG) index, closely associated with the progression of metabolic disorders, has emerged as a reliable surrogate marker for assessing IR (10, 11). Several studies have linked the TyG index to various metabolic, cardiovascular, and cerebrovascular diseases (12–14). However, there is a lack of research in the literature examining the relationship between the TyG index and CHF with AF. This study aimed to investigate the association between the TyG index and the clinical outcome of patients with CHF and AF.

## Methods

### Study population

This study utilized data from the Medical Information Mart for Intensive Care-IV (MIMIC-IV) database, a repository of comprehensive medical records from patients admitted to the intensive care units (ICUs) at Beth Israel Deaconess Medical Center (15). The MIT Computational Physiology Laboratory managed this database. The author (Fuqiang Kan), authorized to access the dataset (ID: 12552525), completed the US National Institutes of Health's online course on protecting human research participants and oversaw data extraction. Adherence to STROBE guidelines for observational research was maintained.

**Figure 1:**
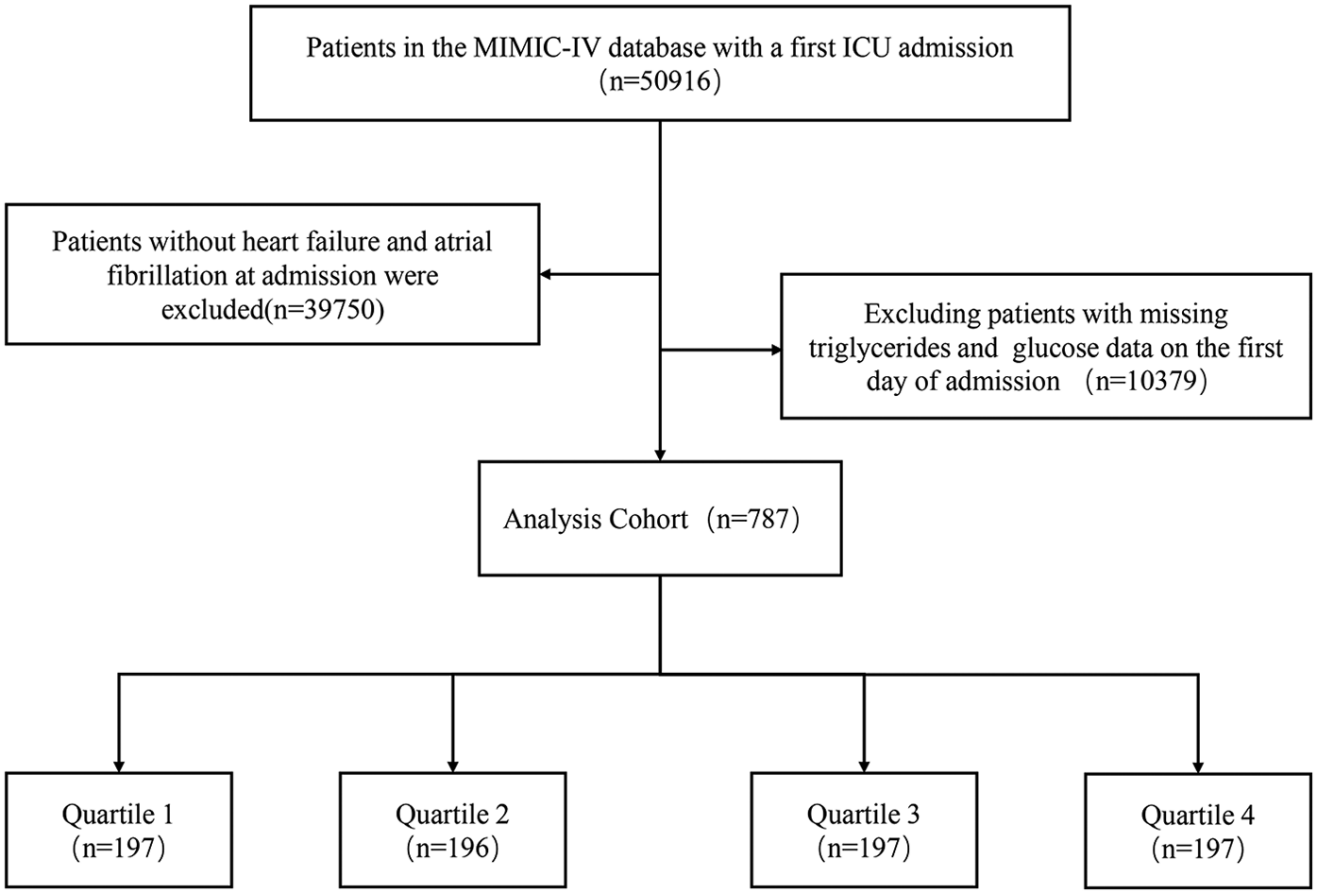
Flowchart of patient selection.

Patients meeting the following criteria were included: (a) Diagnosed with CHF and AF based on the ninth revision of the International Classification of Diseases (ICD-9) code (code 4280 and code 42731); (b) Adults aged 18 and older; (c) First admission to ICUs. Participants lacking TGs and fasting blood glucose (FBG) data on their initial ICU admission day were excluded. A total of 787 patients were ultimately included and categorized into four groups based on their TyG index quartiles on the first ICU admission day (Figure 1).

### Data collection

Data extraction utilized PostgresSQL software (version 16) and Navicat Premium software (version 16.1.15) through Structured Query Language execution.

Variable extraction comprised four main groups: (a) Demographics—age, gender, and race; (b) Comorbidities—Hypertension, diabetes, and chronic kidney disease (CKD); (c) Laboratory indicators—white blood cells (WBCs), red blood cells, hemoglobin, platelets, serum sodium, serum potassium, serum calcium, anion gap, international normalized ratio (INR), urea nitrogen, serum creatinine, total cholesterol (TC), low-density lipoprotein cholesterol (LDL-C), high-density lipoprotein cholesterol (HDL-C), FBG, and TG; (d) Severity of illness scores at admission—Acute Physiology Score III (APSIII), simplified Acute Physiology Score II (SAPS-II), Oxford Acute Severity of Illness Score (OASIS), and Sepsis-related Organ Failure Assessment score (SOFA). It is worth noting that CKD is a well-known risk marker, and in this study, CKD was diagnosed according to the Ninth Revision of the ICD-9 codes (codes 5859). The TyG index was calculated using the formula ln[fastingTGs(mg/dl)×FBG(mg/dl)/2] (16). All variables and severity scores were extracted within the first 24 h of ICU admission.

### Clinical outcomes

The primary focus of this study was hospital mortality, with secondary attention to ICU mortality.

### Statistical analysis

Continuous variables were portrayed as mean ± SD or median (interquartile range) based on data distribution, while categorical variables were presented as proportions. Differences in continuous variables were examined using the analysis of variance test or the rank sum test. For categorical variables, the Chi-square test or Fisher's exact test was employed to compare the characteristics of subjects in the result group.

Kaplan–Meier survival analysis assessed endpoint incidence rates among TyG index groups, with log-rank tests determining differences. Multivariable logistic regression models calculated odds ratios (ORs) and 95% confidence intervals (CIs) between the TyG index and endpoints, adjusting for various models. Confounding variables were selected based on a *P* value <0.05 in univariate analysis. Clinically relevant and prognosis-associated variables were included in the multivariate model: model 1 (unadjusted); model 2 (adjusted for age, gender, and race); model 3 (adjusted for age, gender, race, hypertension, diabetes, CKD, WBC, hemoglobin, serum potassium, serum calcium, anion gap, INR, urea nitrogen, serum creatinine, and LDL).

Additionally, a restricted cubic spline regression model analyzed the nonlinear relationship between the baseline TyG index and hospital and ICU mortality. The TyG index was incorporated into models as continuous or categorical variables, using the first quartile as the reference group. *P* values for trends were calculated based on quartile levels. A stratified analysis, considering age, gender, hypertension, diabetes, and CKD, determined the TyG index's consistency for primary outcomes. Interaction between the TyG index and stratification variables was assessed through the likelihood-ratio test.

This study employed a two-tailed test, considering a *P*-value <0.05 statistically significant. For missing data, a single imputation method based on an iterative imputer was used, employing a Bayesian Ridge model. All statistical analyses were conducted using R Statistical Software (http://www.R-project.org, The R Foundation) and the Free Statistics Analysis Platform (Beijing, China) (17).

## Results

A total of 787 patients were included in this study, with an average age of 73.1 ± 13.6 years, and 419 (53.2%) being male. Among all participants, the average TyG index value was 8.9 ± 0.7. The rates of hospital mortality and ICU mortality were 14.2% and 8.3%, respectively.

### Baseline characteristics

Table 1 presents the baseline characteristics of critically ill patients with CHF and AF categorized by TyG index quartiles. Participants were distributed into four groups based on TyG index levels at admission (Quartiles: Q1, 7.213–8.456; Q2, 8.457–8.835; Q3, 8.841–9.300; and Q4, 9.301–13.493). The mean TyG index values for each quartile were 8.2 ± 0.2, 8.6 ± 0.1, 9.1 ± 0.1, and 9.9 ± 0.6, respectively. Patients in the highest TyG index quartile exhibited a higher prevalence of diabetes and CKD, elevated severity of illness scores at admission, increased WBC levels, TC, urea nitrogen, serum creatinine, and anion gap, as well as lower levels of HDL-C. Additionally, this group showed longer hospital and ICU stays, along with higher rates of hospital and ICU mortality.

**Table 1:** Characteristics and outcomes of participants categorized by TyG index.

Categories	Total (*n* = 787)	Q1 (*n* = 197)	Q2 (*n* = 196)	Q3 (*n* = 197)	Q4 (*n* = 197)	*P*-value
Demographic						
Age (years)	73.1 ± 13.6	76.5 ± 13.8	72.9 ± 13.8	74.3 ± 12.3	68.6 ± 13.2	<0.001
Gender (male)	419 (53.2)	104 (52.8)	106 (54.1)	96 (48.7)	113 (57.4)	0.388
Race						0.827
Black	65 (8.3)	19 (9.6)	18 (9.2)	12 (6.1)	16 (8.1)	
White	639 (81.2)	158 (80.2)	159 (81.1)	167 (84.8)	155 (78.7)	
Asian	22 (2.8)	6 (3)	4 (2)	6 (3)	6 (3)	
Other	61 (7.8)	14 (7.1)	15 (7.7)	12 (6.1)	20 (10.2)	
Laboratory tests						
WBC (K/ul)	10.7 (8.1, 14.0)	9.2 (7.2, 12.1)	10.7 (8.1, 13.6)	11.7 (8.4, 14.4)	11.3 (8.9, 14.8)	<0.001
RBC (K/ul)	3.9 ± 0.7	3.9 ± 0.6	3.9 ± 0.7	3.9 ± 0.7	3.8 ± 0.7	0.318
Platelet (K/ul)	225.3 ± 94.7	212.8 ± 86.1	233.7 ± 105.2	229.7 ± 94.4	224.9 ± 91.5	0.146
Hemoglobin (g/dl)	11.7 ± 2.1	11.8 ± 1.9	11.8 ± 2.1	11.7 ± 2.2	11.4 ± 2.0	0.181
TG (mg/dl)	100.0 (74.0, 147.5)	64.0 (52.0, 75.0)	89.0 (77.0, 105.2)	114.0 (98.0, 141.0)	197.0 (153.0, 278.0)	<0.001
TC (mg/dl)	155.4 ± 48.2	142.7 ± 42.0	155.9 ± 52.8	159.3 ± 43.0	163.5 ± 51.8	<0.001
HDL-C (mg/dl)	44.8 ± 16.5	51.2 ± 17.2	47.5 ± 16.1	44.6 ± 15.4	35.9 ± 13.3	<0.001
LDL-C (mg/dl)	79.0 (56.0, 106.5)	72.0 (56.0, 95.0)	81.0 (64.0, 112.2)	89.0 (61.0, 110.0)	72.0 (48.0, 105.0)	<0.001
Urea nitrogen (mg/dl)	21.0 (15.0, 33.0)	18.5 (14.5, 28.7)	19.2 (14.0, 26.8)	24.0 (16.5, 33.5)	24.5 (16.8, 47.0)	<0.001
Creatinine (mg/dl)	1.0 (0.8, 1.5)	0.9 (0.7, 1.3)	1.0 (0.8, 1.3)	1.0 (0.8, 1.5)	1.2 (0.8, 2.0)	<0.001
Sodium (mEq/L)	138.5 ± 4.2	138.1 ± 4.1	138.8 ± 4.1	138.9 ± 4.2	138.2 ± 4.2	0.108
Potassium (mEq/L)	4.1 ± 0.5	4.1 ± 0.5	4.1 ± 0.5	4.1 ± 0.5	4.1 ± 0.5	0.373
Calcium (mEq/L)	8.5 ± 0.7	8.5 ± 0.6	8.5 ± 0.6	8.5 ± 0.7	8.4 ± 0.9	0.41
Chloride (mEq/L)	103.9 ± 5.4	103.9 ± 4.9	104.3 ± 5.3	104.3 ± 5.6	103.1 ± 5.6	0.108
FBG (mg/dl)	148.0 ± 56.1	113.9 ± 22.3	129.9 ± 29.2	155.0 ± 53.1	193.2 ± 69.6	<0.001
Aniongap (mEq/L)	14.5 ± 3.2	13.9 ± 2.8	14.1 ± 2.7	14.5 ± 2.7	15.7 ± 4.2	<0.001
INR	1.4 ± 0.5	1.4 ± 0.5	1.4 ± 0.6	1.4 ± 0.5	1.4 ± 0.5	0.85
TyG	8.9 ± 0.7	8.2 ± 0.2	8.6 ± 0.1	9.1 ± 0.1	9.9 ± 0.6	<0.001
Comorbidities						
Hypertension	458 (58.2)	123 (62.4)	117 (59.7)	117 (59.4)	101 (51.3)	0.131
Diabetes	263 (33.4)	35 (17.8)	40 (20.4)	74 (37.6)	114 (57.9)	<0.001
CKD	148 (18.8)	34 (17.3)	30 (15.3)	38 (19.3)	46 (23.4)	0.206
GCS	15.0 (13.0, 15.0)	14.0 (13.0, 15.0)	15.0 (13.0, 15.0)	15.0 (12.0, 15.0)	15.0 (14.0, 15.0)	0.066
OASIS	32.0 (27.0, 39.0)	31.0 (26.0, 37.0)	31.0 (25.0, 37.0)	33.0 (27.0, 40.0)	35.0 (30.0, 41.0)	<0.001
SOFA	3.0 (2.0, 6.0)	3.0 (2.0, 4.0)	3.0 (2.0, 5.0)	4.0 (2.0, 6.0)	5.0 (2.0, 9.0)	<0.001
APSIII	41.0 (31.0, 55.0)	37.0 (28.0, 49.0)	39.5 (30.0, 50.2)	42.0 (32.0, 54.0)	48.0 (35.0, 65.0)	<0.001
Outcomes						
LOS ICU (days)	2.6 (1.5, 5.0)	2.0 (1.2, 3.8)	2.5 (1.5, 5.0)	2.8 (1.3, 5.6)	3.2 (1.8, 6.9)	<0.001
LOS hospital (days)	7.2 (4.4, 12.9)	6.0 (4.0, 10.8)	7.2 (4.6, 12.0)	7.9 (4.2, 13.1)	7.7 (4.6, 15.8)	0.069
ICU mortality	65 (8.3)	8 (4.1)	14 (7.1)	16 (8.1)	27 (13.7)	0.006
Hospital mortality	112 (14.2)	17 (8.6)	29 (14.8)	28 (14.2)	38 (19.3)	0.026

TyG index: Q1 (7.213–8.456), Q2 (8.457–8.835), Q3 (8.841–9.300), Q4 (9.301–13.493).

Abbreviations: WBC, white blood cells; RBC, red blood cells; TG, triglycerides; TC, total cholesterol; HDL-C, high-density lipoprotein cholesterol; LDL-C, low-density lipoprotein cholesterol; FBG, fast blood glucose; INR, international normalized ratio; TyG, triglyceride-glucose; CKD, Chronic kidney disease; GCS, Glasgow coma scale; OASIS, Oxford acute severity of illness score; SOFA, sequential organ failure assessment; APSIII, acute physiology score III.

Table 2 illustrates the differences in baseline characteristics between survivors and non-survivors during the hospital stay. Factors associated with hospital mortality (*P* < 0.05) included gender, age, WBCs, hemoglobin, LDL-C, urea nitrogen, serum creatinine, potassium, and calcium. The TyG index levels in the non-survivor group were significantly higher than those in the survivor group (9.2 ± 0.8 vs. 8.9 ± 0.7, *P* < 0.001).

**Table 2:** Baseline characteristics of the survivors and non-survivors groups.

Categories	Total (*n* = 787)	Survivor (*n* = 675)	Non-survivor (*n* = 112)	*P*-value
Demographic				
Age (years)	73.1 ± 13.6	72.6 ± 13.6	76.0 ± 13.0	0.013
Gender (male)	419 (53.2)	370 (54.8)	49 (43.8)	0.03
Race				0.921
Black	65 (8.3)	57 (8.4)	8 (7.1)	
White	639 (81.2)	545 (80.7)	94 (83.9)	
Asian	22 (2.8)	19 (2.8)	3 (2.7)	
Other	61 (7.8)	54 (8)	7 (6.2)	
Laboratory tests				
WBC (K/ul)	10.7 (8.1, 14.0)	10.5 (7.9, 13.6)	12.4 (9.6, 15.7)	<0.001
RBC (K/ul)	3.9 ± 0.7	3.9 ± 0.7	3.8 ± 0.8	0.153
Platelet (K/ul)	225.3 ± 94.7	226.3 ± 95.6	218.8 ± 89.5	0.437
Hemoglobin (g/dl)	11.7 ± 2.1	11.7 ± 2.0	11.2 ± 2.3	0.022
TG (mg/dl)	100.0 (74.0, 147.5)	101.0 (73.5, 146.0)	99.5 (78.8, 154.5)	0.305
TC (mg/dl)	155.4 ± 48.2	156.6 ± 48.3	147.9 ± 47.2	0.076
HDL-C (mg/dl)	44.8 ± 16.5	45.1 ± 17.0	43.2 ± 13.5	0.271
LDL-C (mg/dl)	79.0 (56.0, 106.5)	80.0 (59.0, 107.0)	67.0 (46.0, 100.2)	0.007
Urea nitrogen (mg/dl)	21.0 (15.0, 33.0)	20.7 (15.0, 31.0)	27.3 (18.9, 43.2)	<0.001
Creatinine (mg/dl)	1.0 (0.8, 1.5)	1.0 (0.8, 1.4)	1.3 (0.9, 2.0)	<0.001
Sodium (mEq/L)	138.5 ± 4.2	138.5 ± 4.0	138.8 ± 4.7	0.429
Potassium (mEq/L)	4.1 ± 0.5	4.1 ± 0.5	4.2 ± 0.6	0.006
Calcium (mEq/L)	8.5 ± 0.7	8.5 ± 0.7	8.3 ± 0.8	0.004
Chloride (mEq/L)	103.9 ± 5.4	103.8 ± 5.2	104.6 ± 6.2	0.164
FBG (mg/dl)	148.0 ± 56.1	144.7 ± 54.0	167.9 ± 64.0	<0.001
Aniongap (mEq/L)	14.5 ± 3.2	14.2 ± 2.9	16.5 ± 4.3	<0.001
INR	1.4 ± 0.5	1.4 ± 0.5	1.6 ± 0.7	<0.001
TyG	8.9 ± 0.7	8.9 ± 0.7	9.2 ± 0.8	<0.001
Comorbidities				
Hypertension	458 (58.2)	396 (58.7)	62 (55.4)	0.511
Diabetes	263 (33.4)	228 (33.8)	35 (31.2)	0.599
CKD	148 (18.8)	126 (18.7)	22 (19.6)	0.807
GCS	15.0 (13.0, 15.0)	15.0 (13.0, 15.0)	14.5 (8.8, 15.0)	0.01
OASIS	41.0 (31.0, 55.0)	39.0 (31.0, 51.0)	56.5 (36.0, 78.2)	<0.001
SOFA	32.0 (27.0, 39.0)	32.0 (26.0, 38.0)	40.0 (34.0, 47.0)	<0.001
APSIII	3.0 (2.0, 6.0)	3.0 (2.0, 5.0)	5.5 (3.0, 8.2)	<0.001

Abbreviations: WBC, white blood cells; RBC, red blood cells; TG, triglycerides; TC, total cholesterol; HDL-C, high-density lipoprotein cholesterol; LDL-C, low-density lipoprotein cholesterol; FBG, fast blood glucose; INR, international normalized ratio; TyG, triglyceride-glucose; CKD, chronic kidney disease; GCS, Glasgow coma scale; OASIS, Oxford acute severity of illness score; SOFA, sequential organ failure assessment; APSIII, acute physiology score III**.**

### Kaplan–Meier survival analysis

**Figure 2:**
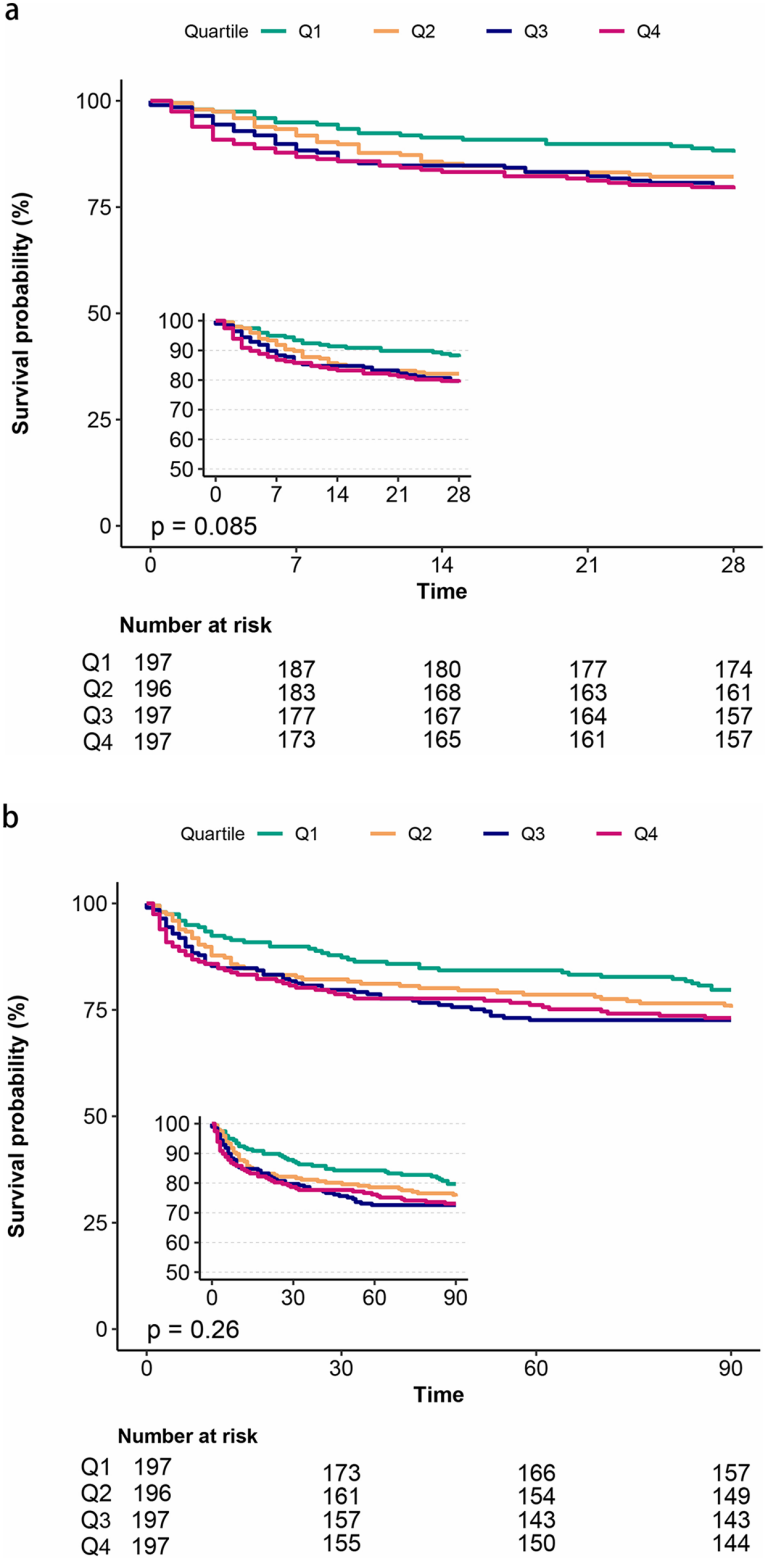
Kaplan–Meier survival analysis curves for all-cause mortality. Kaplan–Meier curves showing cumulative probability of all-cause mortality according to groups at 28 days **(a)**, and 3 months **(b)**.

The Kaplan–Meier survival analysis curves were employed to evaluate the incidence of primary outcomes among different groups, stratified by the quartiles of the TyG index. Figure 2 graphically represents the results. Patients with a higher TyG index exhibited an elevated risk of hospital and ICU mortality. However, no significant difference was observed during the short-term follow-ups of 28 days and 3 months (log-rank *P* = 0.085, 0.26, respectively).

### Multivariable logistic regression analysis

Table 3 outlines the results of the multivariable logistic regression analysis investigating the association between the TyG index and hospital mortality. The results indicated that the TyG index was a significant risk factor in the unadjusted model [OR, 1.54 (95% CI: 1.2–1.98) *P* = 0.001], partly adjusted model [OR, 1.75 (1.33–2.29) *P* < 0.001], and fully adjusted model [OR, 1.59 (1.15–2.19) *P* = 0.005] when considering the TyG index as a continuous variable. When the TyG index was treated as a nominal variable, patients in the higher quartile were significantly associated with a higher risk of hospital death in the three established multiple logistic regression models—unadjusted [OR, 2.53 (1.37–4.66) *P* = 0.003], partly adjusted [OR, 3.18 (1.68–5.99) *P* < 0.001], and fully adjusted models [OR, 2.67 (1.3–5.5) *P* = 0.008]—compared to subjects in the lowest quartile. This trend was consistent with an increase in the TyG index. Similar findings were observed in the multiple logistic regression analysis of the TyG index and ICU mortality.

**Table 3:** Multivariate logistic regression analyses of TyG index and all-cause mortality.

Categories	Model 1		Model 2		Model 3	
	OR_95CI	*P*-value	OR_95CI	*P*-value	OR_95CI	*P*-value
Hospital mortality						
TyG	1.54 (1.2–1.98)	0.001	1.75 (1.33–2.29)	<0.001	1.59 (1.15–2.19)	0.005
Quartile						
Q1 (*n* = 197)	1 (Ref)		1 (Ref)		1 (Ref)	
Q2 (*n* = 196)	1.84 (0.97–3.47)	0.06	2.02 (1.06–3.84)	0.032	2.22 (1.12–4.38)	0.022
Q3 (*n* = 197)	1.75 (0.93–3.32)	0.084	1.85 (0.97–3.53)	0.062	2.02 (1.01–4.04)	0.047
Q4 (*n* = 197)	2.53 (1.37–4.66)	0.003	3.18 (1.68–5.99)	<0.001	2.67 (1.3–5.5)	0.008
*P* for trend		0.005		0.001		0.015
ICU mortality						
TyG	1.75 (1.3–2.35)	<0.001	1.87 (1.37–2.57)	<0.001	1.9 (1.28–2.83)	0.001
Quartile						
Q1 (*n* = 197)	1 (Ref)		1 (Ref)		1 (Ref)	
Q2 (*n* = 196)	1.82 (0.74–4.43)	0.189	1.92 (0.78–4.7)	0.155	2.1 (0.81–5.45)	0.127
Q3 (*n* = 197)	2.09 (0.87–5)	0.098	2.18 (0.91–5.24)	0.082	2.33 (0.91–5.96)	0.078
Q4 (*n* = 197)	3.75 (1.66–8.48)	0.001	4.27 (1.85–9.85)	0.001	3.89 (1.5–10.07)	0.005
*P* for trend		0.001		<0.001		0.006

Model 1: unadjusted.

Model 2: adjusted for age, gender, race.

Model 3: adjusted for age, gender, race, hypertension, diabetes, CKD, WBC, hemoglobin, LDL, urea nitrogen, creatinine, potassium, calcium, anion gap, INR**.**

### Restricted cubic spline regression model

**Figure 3:**
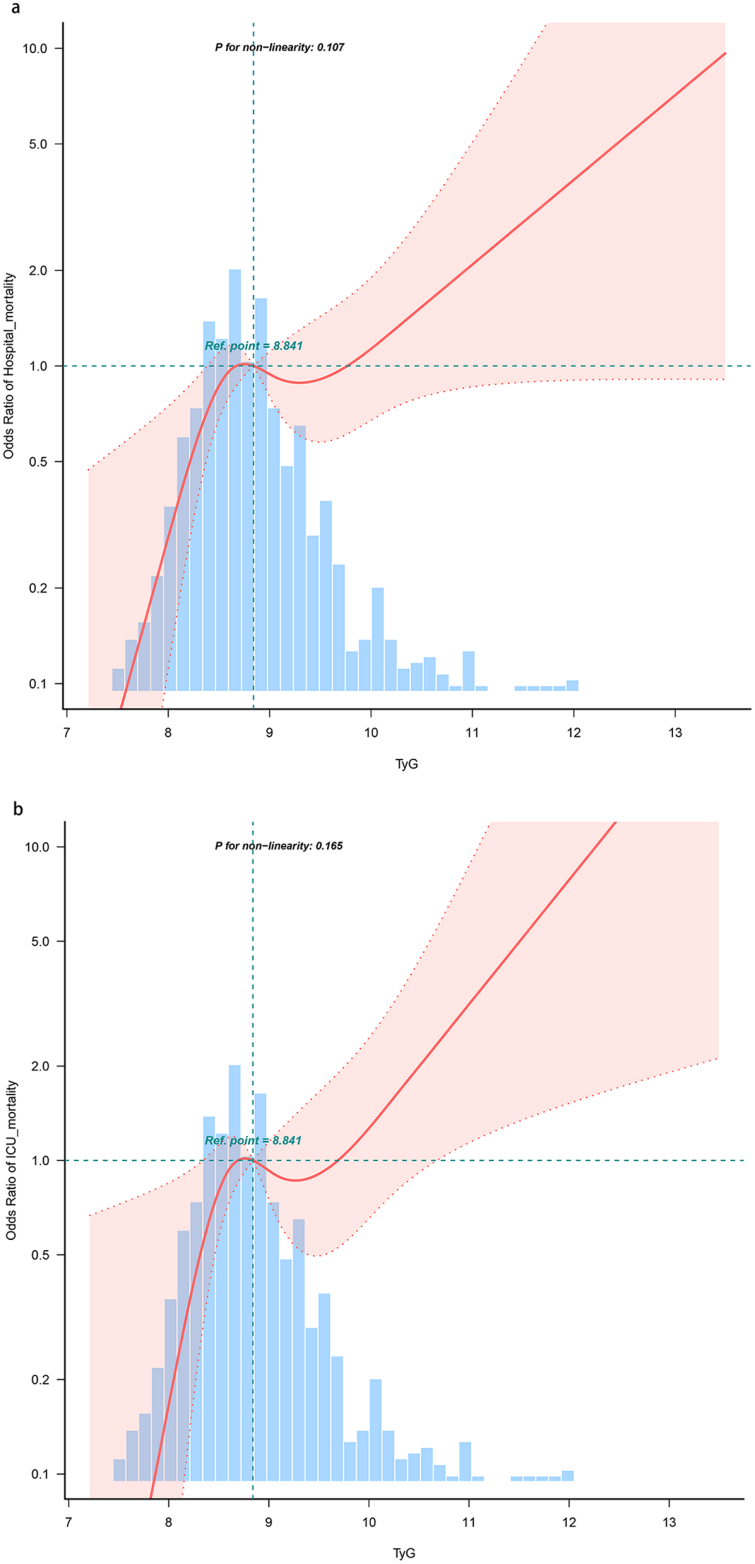
Restricted cubic spline curve for the TyG index and all-cause mortality. **(a)** Restricted cubic spline for hospital mortality. **(b)** Restricted cubic spline for ICU mortality. OR, odds ratio; ICU, intensive care unit; TyG, triglyceride-glucose.

The risk of hospital mortality and ICU mortality increased linearly with the growth of the TyG index (*P* for non-linearity = 0.107 and *P* for non-linearity = 0.165, respectively) (Figure 3).

### Subgroup analysis

**Figure 4:**
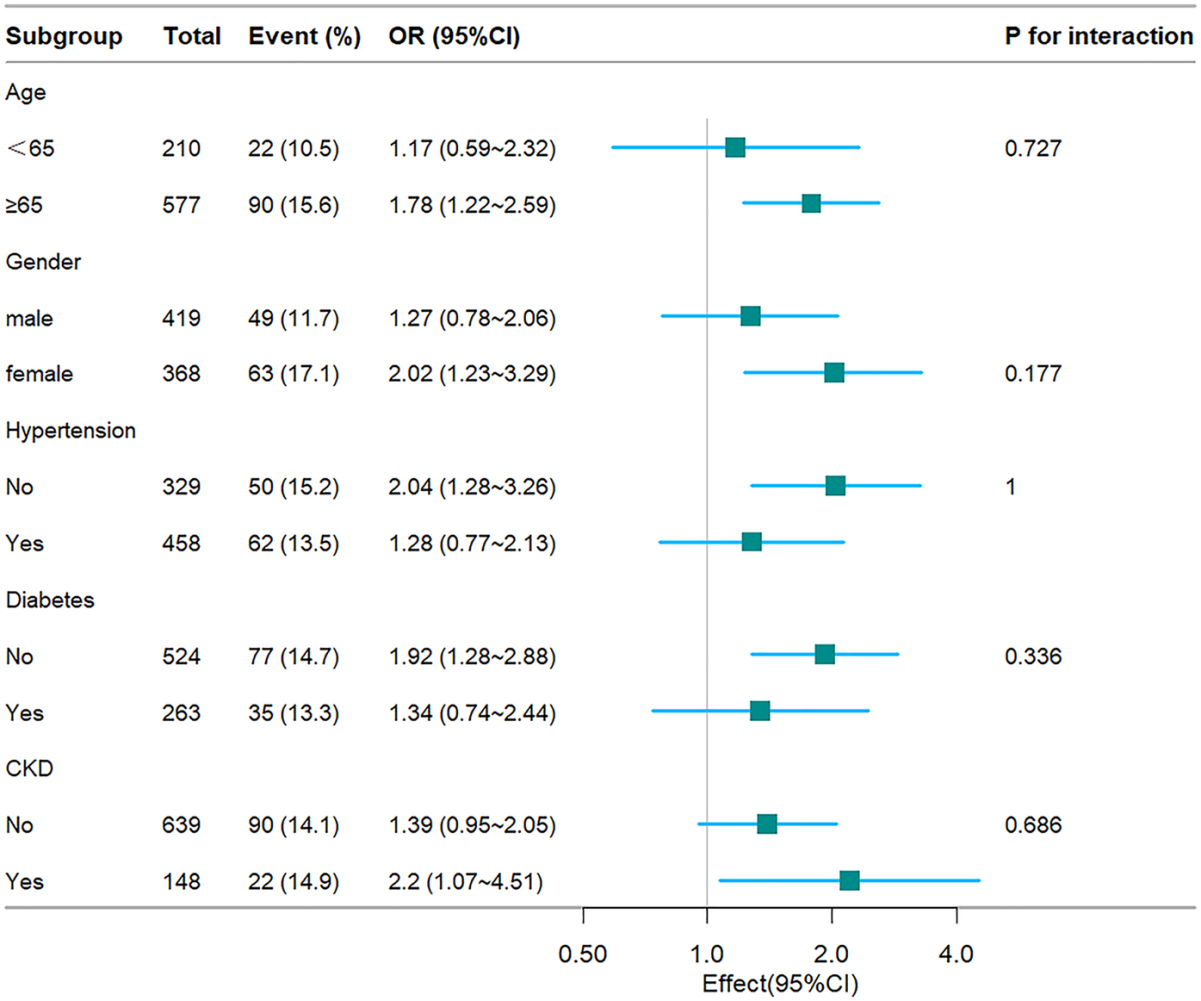
Fores plots of odds ratios for the hospital mortality in different subgroups. OR, odds ratio; CI, confidence interval; CKD, chronic kidney disease.

**Figure 5:**
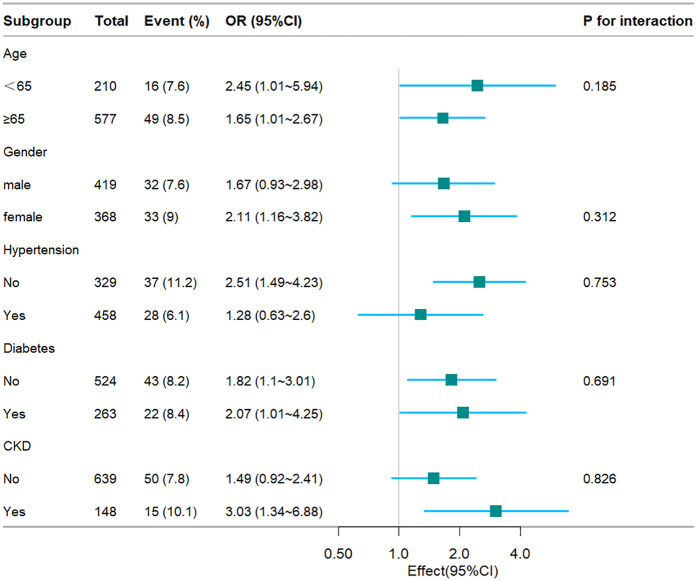
Fores plots of odds ratios for the ICU mortality in different subgroups. OR, odds ratio; CI, confidence interval; CKD, chronic kidney disease.

To further substantiate the relationship between the TyG index and hospital mortality, ICU mortality, stratified analyses were conducted based on age, gender, hypertension, diabetes, and CKD (see Figures 4, 5). The TyG index exhibited a significant association with a higher risk of hospital mortality in CHF and AF patient subgroups, including females [OR (95% CI) 2.02 (1.23–3.29)], those aged 65 years or older [OR (95% CI) 1.78 (1.22–2.59)], those without hypertension [OR (95% CI) 2.04 (1.28–3.26)], those without diabetes [OR (95% CI) 1.92 (1.28–2.88)], and those with CKD [OR (95% CI) 2.2 (1.07–4.51)]. Similarly, concerning stratified analyses of ICU mortality, the TyG index demonstrated a significant association with a higher risk of ICU mortality in subgroups, including females [OR (95% CI) 2.11 (1.16–3.82)], those aged <65 years [OR (95% CI) 2.45 (1.01–5.94)], those aged 65 years or older [OR (95% CI) 1.65 (1.01–2.67)], those without hypertension [OR (95% CI) 2.51 (1.49–4.23)], those with diabetes [OR (95% CI) 2.07 (1.01–4.25)], those without diabetes [OR (95% CI) 1.82 (1.1–3.01)], and those with CKD [OR (95% CI) 3.03 (1.34–6.88)]. No interactions were observed between the TyG index and age, gender, hypertension, diabetes, and CKD in subgroup analyses (all *P* values for interaction >0.05).

## Discussion

To the best of our knowledge, this study is the first to explore the relationship between the TyG index and all-cause mortality in patients with CHF and AF. Our findings revealed a significant association between an elevated TyG index and increased all-cause ICU and hospital mortality among patients with CHF and AF. Importantly, this association remained robust even after adjusting for multiple clinical and laboratory variables, suggesting that the TyG index could be a valuable decision-making tool for clinicians managing patients with CHF and AF.

In recent years, the TyG index has emerged as a potential indicator of metabolic disorders, type 2 diabetes mellitus, atherosclerotic disease, cerebrovascular disease, and cardiovascular disease (CVD) (12–14, 18, 19). Numerous clinical studies have explored the link between an elevated TyG index and higher morbidity and mortality in the general population or other patient cohorts (20, 21). According to Liu et al., an increased TyG index is associated with coronary artery disease, myocardial infarction, and CVD in the general population (22). Zheng's research indicated that a high cumulative TyG index is linked to a higher risk of HF (23). Yang et al. reported that the TyG index may serve as a predictive marker for adverse cardiovascular outcomes in patients with chronic coronary syndrome (24). Multiple meta-analyses suggest that the TyG index is associated with various coronary artery diseases (25–27). Chen et al. observed that an elevated TyG index is an independent risk factor for AF among non-diabetic hospitalized patients (28). And Alireza Azarboo et al. found in their research that the TyG index is an easy-to-measure surrogate marker of IR in patients with AF (29). Another study involving 1,226 patients suggested that an elevated TyG index was associated with an increased risk of stroke recurrence and death (30). Similar results are also observed in the studies of Yang et al. (31). A meta-analysis has identified a significant association between TyG levels and Peripheral artery disease (PAD) and its severity (32). Collectively, these studies underscore the potential of the TyG index in predicting clinical outcomes related to cerebrovascular diseases and CVDs.

Our findings suggest a significant association between a high TyG index and all-cause mortality in patients with CHF and AF. Upon considering and adjusting for various covariates, a stronger correlation is observed. However, the precise biological mechanism underlying the relationship between the TyG index, CHF, and AF remains uncertain. A potential mechanism could be associated with IR. Hyperinsulinemic-euglycemic Clamp is the gold standard for diagnosing insulin resistance (IR), but due to its limitations, it is difficult to apply in large-scale clinical studies. HOMA-IR is the most widely used surrogate marker; however, its calculation requires the measurement of fasting insulin concentrations (33). The TyG index serves as a simple and widely used method for determining IR (11). Previous studies have demonstrated that compared to the hyperinsulinemic-euglycemic Clamp technique, the TyG index exhibits good sensitivity and specificity in diagnosing IR (33). Extensive research has demonstrated the intimate association of IR with endothelial dysfunction, oxidative stress, immune deregulation, coagulation imbalance, and inflammatory responses (34–36). The state of IR can lead to a significant accumulation of fatty acids and TGs within cardiomyocytes, giving rise to “cardiac lipotoxicity.” This condition has the potential to trigger cellular dysfunction, cardiomyocyte apoptosis, and hinder myocardial metabolism, ultimately altering the function and structure of cardiomyocytes and elevating the risk of HF and arrhythmias (37–39). Our research revealed that the different TyG index quartiles reflect consistent variations of inflammatory and metabolic markers, such as WBC, hemoglobin, and creatinine. Consequently, we hypothesize that these parameters may also drive the results, albeit the underlying mechanisms are yet to be elucidated and require further exploration through fundamental research.

In sensitivity analysis, the consistent direction of all results indicates the stability and reliability of the core outcomes. Furthermore, our study found that the linear relationship between the TyG index and all-cause mortality in patients with CHF and AF remained steady in those without diabetes and non-hypertensive individuals. This result might be attributed to the higher likelihood of patients with these comorbidities adhering to appropriate treatment regimens and adopting healthier lifestyle habits (40). Additionally, our study revealed that the association between the TyG index and all-cause mortality seemed to be more pronounced in patients with CKD. CKD is a frequent comorbidity in HF, and worsening renal function often accompanies HF decompensation. Similarly, there is a bidirectional relationship between AF and CKD (41, 42). Patients with HF and AF usually require anticoagulation therapy, but severe renal dysfunction limits the use of such therapy (41). To some extent, this limitation may contribute to an increase in all-cause mortality. In summary, the TyG index emerges as a significant predictor of all-cause mortality in patients with CHF and AF.

However, our study comes with several limitations. Firstly, the results obtained may not be entirely representative as the enrolled patients were from an American population. The inherent limitations of public databases introduce the possibility of unmeasured variables, such as genetic factors, dietary patterns, and psychosocial factors. Secondly, our analysis only considered the baseline TyG index. To comprehensively understand its prognostic significance, observing the dynamics of the TyG index over time in patients with CHF and AF is imperative. This longitudinal approach would offer a more complete understanding of the index's predictive value. Thirdly, our study employed a retrospective analysis from an observational study, lacking direct evidence to firmly establish a causal relationship. Nevertheless, we applied a comprehensive array of rigorous statistical methods to ensure the robustness and credibility of our findings. Fourthly, as this study is a retrospective analysis, it is not feasible to differentiate patients with paroxysmal, persistent, or permanent AF, or those with or without AF upon admission. Additionally, the same limitation extends to differentiating HF with reduced EF (HFrEF), HF with mid-range EF (HFmrEF), and HF with preserved EF (HFpEF) among patients diagnosed with CHF. In conclusion, while our study demonstrated the prognostic significance of the TyG index in patients with CHF and AF, its practical clinical utility requires validation through larger-scale prospective studies.

## Conclusions

In summary, our results suggest a strong association between an elevated TyG index and increased hospital mortality and ICU mortality in patients with CHF and AF. Monitoring the TyG index holds the potential to inform clinical decision-making and enhance disease management in clinical practice. However, further prospective studies are necessary to validate these findings.

## Data Availability

The original contributions presented in the study are included in the article/Supplementary Material, further inquiries can be directed to the corresponding author.
